# Characterization of the gut microbiota of Kawasaki disease patients by metagenomic analysis

**DOI:** 10.3389/fmicb.2015.00824

**Published:** 2015-08-11

**Authors:** Akiko Kinumaki, Tsuyoshi Sekizuka, Hiromichi Hamada, Kengo Kato, Akifumi Yamashita, Makoto Kuroda

**Affiliations:** ^1^Department of Pediatrics, Graduate School of Medicine, University of TokyoBunkyo-ku, Japan; ^2^Laboratory of Bacterial Genomics, Pathogen Genomics Center, National Institute of Infectious DiseasesShinjuku-ku, Japan; ^3^Department of Pediatrics, Faculty of Medicine, Yachiyo Medical Center, Tokyo Women's Medical UniversityYachiyo, Japan

**Keywords:** Kawasaki disease, gut microbiota, metagenomic analysis, *Streptococcus*, mitis group

## Abstract

Kawasaki disease (KD) is an acute febrile illness of early childhood. Previous reports have suggested that genetic disease susceptibility factors, together with a triggering infectious agent, could be involved in KD pathogenesis; however, the precise etiology of this disease remains unknown. Additionally, previous culture-based studies have suggested a possible role of intestinal microbiota in KD pathogenesis. In this study, we performed metagenomic analysis to comprehensively assess the longitudinal variation in the intestinal microbiota of 28 KD patients. Several notable bacterial genera were commonly extracted during the acute phase, whereas a relative increase in the number of *Ruminococcus* bacteria was observed during the non-acute phase of KD. The metagenomic analysis results based on bacterial species classification suggested that the number of sequencing reads with similarity to five *Streptococcus* spp. (*S. pneumonia, pseudopneumoniae, oralis, gordonii*, and *sanguinis*), in addition to patient-derived *Streptococcus* isolates, markedly increased during the acute phase in most patients. *Streptococci* include a variety of pathogenic bacteria and probiotic bacteria that promote human health; therefore, this further species discrimination could comprehensively illuminate the KD-associated microbiota. The findings of this study suggest that KD-related *Streptococci* might be involved in the pathogenesis of this disease.

## Introduction

Kawasaki disease (KD) is an acute febrile illness of early childhood. The principal pathology is systemic vasculitis with coronary artery involvement, and KD is the leading cause of acquired heart disease in developed countries. It was originally described by Dr. Tomisaku Kawasaki in 1967 (Kawasaki, 1967), and it is known to occur worldwide in children of all races. However, as the etiology of KD remains unknown, no specific biological markers for diagnostic testing have been characterized to date. The diagnosis of KD is based on the following six clinical features: fever lasting for at least 5 days, changes in the extremities, polymorphous exanthem, bilateral conjunctival injection without exudate, changes in the lips and oral cavity, and cervical lymphadenopathy (Newburger et al., 2004). Although the simultaneous intravenous infusion of gamma globulin and aspirin is effective in reducing systemic inflammation and preventing coronary artery involvement, coronary abnormalities still develop in ~5% of affected children, and some patients show no response to this therapy (Newburger et al., 1991).

The annual incidence of KD is increasing rapidly in Japan, with 239.6/100,000 children under the age of 5 years affected in 2010. This incidence is by far the highest rate worldwide (Nakamura et al., 2012), and the risk of KD in siblings of affected children is significantly higher than that in the general population (Fujita et al., 1989). The annual incidence rates are also relatively high in other East Asian countries (with 113.1/100,000 children under the age of 5 years affected in Korea and 69/100,000 in Taiwan) but are low in Europe and North America (with 4.9–15.2/100,000 children under the age of 5 years affected in European countries and 19–26.2/100,000 in North American countries) (Uehara and Belay, 2012). A higher rate of KD has been reported in Hawaiian children of Japanese descent compared with those of European descent (Holman et al., 2010), suggesting the importance of genetic factors in disease susceptibility.

Epidemiological studies have shown that the age-specific incidence rate of KD is the highest among children aged 6–11 months and that 88.4% of KD patients are less than 1 year of age (Uehara and Belay, 2012). Interestingly, a seasonal variation in the number of affected KD patients has been observed (Nakamura et al., 2008). These findings suggest that an infectious agent may trigger this disease; however, its etiology remains unknown. A GWAS of KD in Japanese patients has revealed susceptibility loci related to immune disorders and a human leukocyte antigen; such extensive studies will facilitate characterization of the pathogenesis and pathophysiology (Onouchi, 2012; Onouchi et al., 2012).

Previous reports have suggested that an elevation in lipopolysaccharide (LPS, endotoxin)-binding neutrophils or plasma proteins (Takeshita et al., 1999, 2002b), antibody reactivity against mycobacterial heat-shock protein (HSP65) in convalescent sera (Yokota et al., 1993), and unique TCR Vβ expansion by certain superantigens (SAg) in KD patients (Abe et al., 1992; Yoshioka et al., 1999) might be involved in KD pathogenesis. Case reports have suggested that these factors are attributed to the presence of secondary infections with various pathogens, including *Streptococcus pyogenes, Staphylococcus aureus, Mycoplasma pneumoniae, Chlamydia pneumoniae, Klebsiella pneumoniae*, adenovirus, Epstein-Barr virus, parvovirus B19, herpesvirus 6, parainfluenza virus, measles, rotavirus, dengue virus, varicella zoster virus, cytomegalovirus, and influenza virus (Johnson and Azimi, 1985; Catalano-Pons et al., 2005; Wang et al., 2005; Joshi et al., 2011; Principi et al., 2013). In animal models, exposure to the *Lactobacillus* bacterial cell wall (Duong et al., 2003), immunization with bacillus Calmette-Guérin (BCG) (Nakamura et al., 2007), or exposure to the *Candida albicans* water-soluble fraction (Nagi-Miura et al., 2004; Ohno, 2004) has been shown to induce vasculitis and coronary arteritis. These observations further suggest that infectious agents promote the onset of KD.

The intestinal microbiota constitutes a vast ecosystem with a crucial role in establishing the mucosal immune system, and the intestinal microbiota of healthy adults is considered to be inter-individually variable and intra-individually stable over long time periods (Eckburg et al., 2005; Jakobsson et al., 2010; Arumugam et al., 2011; Jalanka-Tuovinen et al., 2011). By contrast, the intestinal microbiota of infants is different from that of adults, with intestinal microbiota succession being affected by breast or formula feeding, weaning, diet, and unexpected life events, including infection and antibiotic treatment (Stark and Lee, 1982; Palmer et al., 2007; De Filippo et al., 2010; Koenig et al., 2011; Morotomi et al., 2011). The pathogenesis of KD has been suggested to involve a hyperimmune reaction in children who are genetically susceptible to variations in the normal flora; these variations are induced by environmental factors (Lee et al., 2007).

The intestinal microbiota of KD patients is characterized by a lack of *Lactobacilli* during the acute phase (Takeshita et al., 2002a) and the presence of HSP60-producing Gram-negative microbes (genera *Acinetobacter, Enterobacter, Neisseria*, and *Veillonella*) and Gram-positive cocci (genera *Streptococcus* and *Staphylococcus*) with the ability to induce Vβ2 T cell expansion (Nagata et al., 2009). However, these studies on the intestinal profiles of KD patients were performed using culture-based methods.

Metagenomic analyses can reveal both the bacterial and viral compositions of the intestinal microbiota; thus, metagenomics can be used to identify potential pathogens in infectious diseases of unknown etiology (Kuroda et al., 2012). For instance, a metagenomic approach has revealed the presence of *Streptococcus* spp. in lymph node specimens of a KD patient, highlighting the possible role of these bacteria in KD (Katano et al., 2012).

In this study, a comparative metagenomic approach was used to characterize the differential microbiota compositions of KD patients by studying individual clinical specimens in a longitudinal manner. No study to date has performed longitudinal analysis of the microbial microbiota compositions of KD patients using a metagenomic approach. Indeed, although previous studies have suggested a possible role of the intestinal microbiota in the pathogenesis of KD, they have relied only on culture-based methods for microbial detection (Takeshita et al., 2002a; Nagata et al., 2009). We therefore performed metagenomic analysis using a non-culture-based method to expand upon these results.

## Materials and methods

### Clinical specimens used for comparative metagenomic analysis

For the KD patient group, fecal samples were obtained at the time of admission (the acute phase), at the time of discharge (the convalescent phase), and at 4–6 months after the onset of KD (the non-acute phase). The study protocol was approved by the institutional medical ethics committee of the University of Tokyo, Tokyo Women's Medical University and the National Institute of Infectious Diseases in Japan (Approval No. 295), and it was conducted according to the Declaration of Helsinki Principles. Written informed consent was obtained from the parents of all children for publication of their individual details and accompanying images in this manuscript. The consent form is held by the authors' institution and is available for review.

### DNA extraction from fecal samples

Total DNA extraction was performed using a QIAamp® DNA Stool Mini Kit (QIAGEN, Tokyo, Japan) according to the manufacturer's instructions. To increase the recovery of bacterial DNA, particularly from Gram-positive bacteria, pretreatment with lytic enzymes was performed prior to extraction using the stool kit. Briefly, 100 mg of fecal sample was suspended in 10 mL of Tris-EDTA buffer (pH 7.5), and 50 μL of 100 mg/mL lysozyme type VI purified from chicken egg white (MPBIO, Derby, UK) and 50 μL of 1 mg/mL purified achromopeptidase (Wako, Osaka, Japan) were added. The solution was incubated at 37°C for 1 h with mixing, 0.12 g of sodium dodecyl sulfate (final conc. 1%) was added, and the suspension was mixed until it became clear. Next, 100 μL of 20 mg/mL proteinase K (Wako) was added, followed by incubation at 55°C for 1 h with mixing. The cell lysate was then subjected to ethanol precipitation. The precipitant was dissolved in 1.6 mL of ASL buffer from the stool kit and subsequently purified using a QIAamp® DNA Stool Mini Kit (QIAGEN).

### DNA library preparation for metagenomic analysis and short-read DNA sequencing

A DNA library was prepared using a Nextera™ DNA Sample Prep Kit (Illumina-compatible, EPICENTRE Biotechnologies, Madison, WI, USA), and DNA clusters were generated on a slide using a Cluster Generation Kit (version 2) with an Illumina cluster station (Illumina, San Diego, CA, USA) according to the manufacturer's instructions. The general procedure described in the standard protocol (Illumina) was performed to obtain standard ~1.0 × 10^7^ short reads for 1 lane. All of the sequencing runs for generating 126-mers were performed with a Genome Analyzer IIx using an Illumina Sequencing Kit (http://www.illumina.com/systems/retired_gaiix/gaiix-kits.html). Fluorescence images were analyzed using Illumina base-calling pipeline (version 1.4.0) to obtain FASTQ-formatted sequence data. The short-read sequences have been deposited in DNA Data Bank of Japan (DDBJ; accession numbers: DRA000895 and DRA001171). All of the obtained DNA sequencing reads were aligned to a reference human genomic sequence using BWA-SW read-mapping software (Li and Durbin, 2010), with quality trimming to remove low-quality reads. The remaining sequence reads were subjected to a megaBLAST search against a nucleotide database. The results of this search were analyzed and visualized using MEGAN version 4.62.3 (Huson et al., 2011), with a minimum support of 1 hit and a minimum score of 150.

### Principal component analysis (PCA) and PERMANOVA analysis

The sequenced reads were assigned to a taxonomic hierarchy using MEGAN software following a megaBLAST homology search. The raw read counts were normalized by the total number of reads, and then PCA was performed using the R “prcomp” and “plot” functions. Permutational multivariate analysis of variance (PERMANOVA) with “ADONIS” was performed using 10,000 × permutations and the “bray” method with R's vegan package (Anderson, 2001).

### Linear discriminant analysis (LDA) coupled with effect size measurements (LEfSe)

A metagenomic biomarker discovery approach, LEfSe, was used to identify the microbial components whose sequences were more abundant in the fecal samples of the KD patients during the acute phase than in those of the KD patients during the non-acute phase and the controls. For LEfSe, Kruskal–Wallis and pairwise Wilcoxon tests are performed, followed by LDA to assess the effect size of each differentially abundant taxon (Segata et al., 2011). In this study, a *p*-value of <0.05 was considered significant for both statistical methods. Bacteria with markedly increased numbers were defined as those with an LDA score (log_10_) of over 2. Less than 0.01% of the total bacterial reads, corresponding with ≤10^7^ CFU/g feces, were omitted from further analysis because of low and unreliable read counts, although significant LDA scores were observed in LEfSe.

### Isolation of *Streptococcus* spp. and species determination based on 16S-rRNA gene

Cultivation of *Streptococcus* spp. was performed using phenylethyl alcohol agar with 5% sheep blood or chocolate agar under anaerobic conditions at 37°C for 48 h. The bacterial species present were determined by performing 16S-rRNA gene sequencing using the bacterial forward primer Bac27F (5′-AGAGTTTGGATCMTGGCTCAG-3′) and the universal reverse primer Univ1492R (5′-CGGTTACCTTGTTACGACTT-3′) (Eden et al., 1991). The obtained sequences were searched against SILVA ribosomal RNA gene database to identify the bacterial species (Quast et al., 2013).

### Whole-genome and phylogenetic analyses of identified *Streptococcus* spp.

A draft genome sequence was obtained by whole-genome sequencing using MiSeq with a NEXTERA XT library preparation kit (Illumina), followed by *de novo* assembly with A5-MiSeq pipeline (Tritt et al., 2012). The resulting scaffolds were annotated using RAST server (Aziz et al., 2008). Maximum likelihood phylogenetic analysis of *Streptococcus* 16S-rDNA was performed using MEGA 6.0 with 1000 bootstrap iterations (Tamura et al., 2013).

### Minimum inhibitory concentration (MIC) testing

MIC testing was performed using an Etest (bioMerieux, France) on Muller-Hinton agar (Difco, Augsburg), according to CLSI guidelines (CLSI, 2013).

## Results

### KD patients included in comparative metagenomic analysis

This study evaluated 28 KD patients (15 males and 13 females, aged 1–114 mo; median of 25 mo). All of these patients were enrolled within 4 days of the onset of illness, with day 1 defined as the first day of fever, and they all met the diagnostic criteria for KD established by the American Heart Association (Newburger et al., 2004). All of the KD patients in the study received intravenous gamma globulin (2 g/kg) and aspirin (30–50 mg/kg/day). One male patient (patient P2) had a persistent fever despite receiving these therapies and was administered additional intravenous gamma globulin (1 g/kg) and prednisolone sodium succinate (2 mg/kg/day). This patient had transient dilatation of the coronary artery, whereas the other 27 patients showed no evidence of cardiac abnormalities.

In this study, the time of admission was defined as the acute phase, while 4–6 months after the onset of KD was considered the non-acute phase. The profiles of the participants, including the age, sex, concomitant symptoms and empirical antimicrobial treatment received, are shown in Table 1.

**Table 1:** **Kawasaki disease patient information**.

**KD patient**	**Age**	**Sex**	**Concomitant symptoms**	**Antimicrobial treatment during the acute phase**
P1	3 y 11 m	M	Vomiting, cough	CMZ
P2	2 y 08 m	M	Diarrhea, vomiting, cough	CFPN-PI/CTX
P3	3 y 00 m	F	Cough	CMZ
P4	2 y 01 m	M	Diarrhea	CFPN-PI/CMZ
P5	1 y 03 m	F	–	AZM/CMZ
P6	2 y 03 m	F	Diarrhea	ABPC/CMZ
P7	3 y 04 m	F	Diarrhea, vomiting	CFPN-PI/CMZ
P8	0 y 08 m	F	Diarrhea, vomiting	ABPC-CVA/CMZ
P9	2 y 06 m	M	Vomiting, abdominal pain	CPDX-PR
P10	5 y 06 m	M	Cough	–
P11	7 y 05 m	F	Diarrhea	CAM
P12	2 y 10 m	F	Neck stiffness	–
P13	0 y 03 m	M	–	CCL
P14	2 y 02 m	M	Rhinorrhea	–
P15	1 y 06 m	F	Cough	CFPN-PI
P16	3 y 10 m	M	–	CDTR-PI
P17	0 y 10 m	F	–	–
P18	1 y 06 m	M	–	CFDN
P19	1 y 04 m	M	Cough	CDTR-PI/FOM
P20	2 y 00 m	F	–	–
P21	4 y 04 m	F	Cough, diarrhea	CFPN-PI/TFLX
P22	2 y 09 m	F	–	AMPC
P23	6 y 01 m	M	–	CFPN-PI
P24	3 y 00 m	M	–	CDTR-PI
P25	1 y 11 m	M	–	CFPN-PI/CAM
P26	9 y 06 m	F	–	CDTR-PI
P27	2 y 11 m	M	–	–
P28	0 y 04 m	M	Vomiting	CDTR-PI

y, year; m, month. M, male; F, female. ABPC, ampicillin; ABPC-CVA, ampicillin-clavulanic acid; AMPC, amoxicillin; AZM, azithromycin; CAM, clarithromycin; CCL, cefaclor; CDTR-PI, cefditoren, pivoxil; CFDN, cefdinir; CFPN-PI, cefcapene pivoxil; CMZ, cefmetazole; CPDX-PR, cefpodoxime proxetil; CTX, cefotaxime; FOM, fosfomycin; TFLX, tosufloxacin.

### Gut microbiota analysis comparing the acute and non-acute phases in KD patients

**Figure 1:**
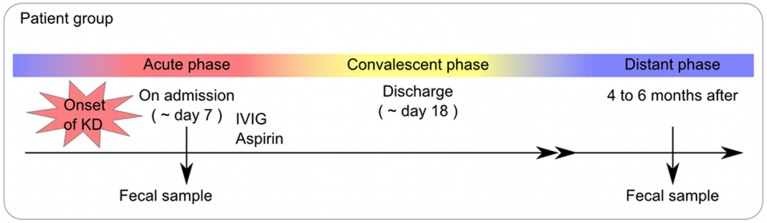
**The protocol for collection of clinical specimens**.

**Figure 2:**
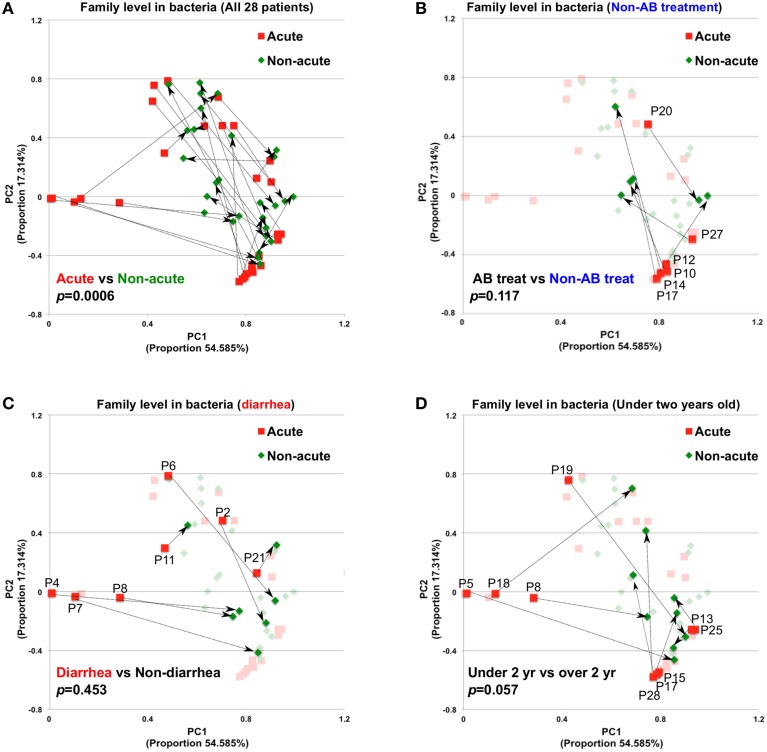
**Principal component analysis of gut microbiota compositions during the acute and non-acute phases of KD. (A)** Relative abundance was estimated at the family taxonomic level (megaBLAST homology score threshold: ≥150). The arrows indicate the corresponding pair for each patient for the acute and non-acute phases (*n* = 28). Some PCA plot components were highlighted based on the indicated patient-related information, group of non-antimicrobial treatment **(B)**, diarrhea symptom **(C)**, and under two years old **(D)**. To investigate the significance between the tested groups, PERMANOVA with “ADONIS” was performed using 10,000 × permutations, in addition to the “bray” method, with R's vegan package.

A total of 56 samples (28 samples each for the acute and non-acute phases) were collected, including two samples from each KD patient (Figure 1 and Table 1). Extracted DNA was subjected to metagenomic sequencing using an Illumina GAIIx next-generation DNA sequencer, and more than 10 million short 126-mer reads were obtained for each specimen. The short reads were classified at the family level of bacteria, with a threshold megaBLAST homology score of = 150. Principal component analysis (PCA) was performed to elucidate the variations between the acute and non-acute phases of KD. The results suggested that the gut microbiota was more variable during the acute phase than during the non-acute phase based on family-level taxonomy (Figure 2A). PERMANOVA with 10,000 × permutations revealed significant dissimilarity of the bacterial communities at the family level between the acute and non-acute phases of KD (*F*-test = 3.7307, *p* = 0.0006) (Figure 2A). The components were highlighted based on the antimicrobial treatment, occurrence of diarrhea, and age group, indicating that such variations during the acute phase might be associated with patient-related factors. Further, the no antimicrobial treatment group during the acute phase had was clustered in the lower right area of the PCA plot (Figure 2B), whereas the diarrhea-positive group during the acute phase exhibited a relatively scattered cluster in the PCA plot (Figure 2C); however, both groups during the non-acute phase were clustered together at the relative center of the plot (Figures 2B,C). The patients in the antimicrobial treatment group did not always exhibit diarrhea symptoms (8 diarrhea/22 antimicrobial treatment), and PERMANOVA indicated no significant differences between the two subject groups, suggesting that the gut microbiota was not affected during the acute phase of KD, regardless of the presence of antimicrobial treatment or diarrhea. The age factor showed a possible association with gut microbiota composition because the *p*-value was close to 0.05 when comparing subjects who were less than 2 years of age with those who were over 2 years of age (Figure 2D).

**Figure 3:**
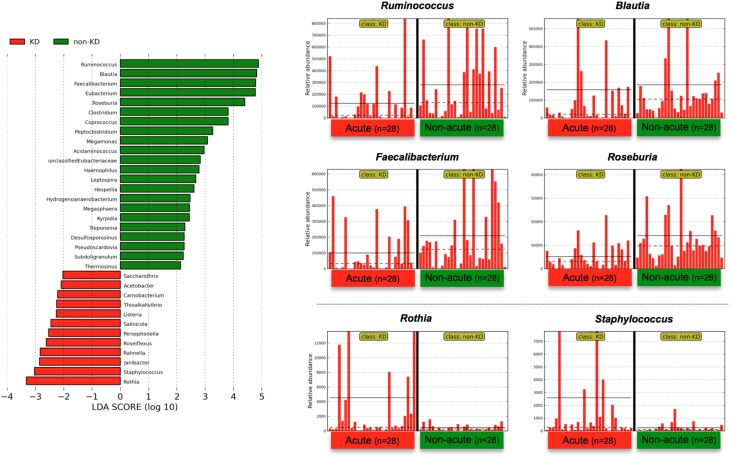
**LEfSe at the genus level**. Linear discriminant analysis (LDA) combined with effect size measurements (LEfSe) revealed a list of features that enable discrimination between the acute and non-acute phases in the fecal samples. A *p*-value of < 0.05 and a score ≥ 2.0 were considered significant in Kruskal–Wallis and pairwise Wilcoxon tests, respectively. The horizontal straight line in the panel indicates the group means, and the dotted line indicates the group medians. The genus *Ruminococcus* was identified as the most predominant during the non-acute phase, and the number of detected reads for all 28 patients was plotted in bar form in the upper-right panel. The straight line indicates the group means, and the dotted line indicates the group medians. Less than 0.01% of the total bacterial reads, corresponding to ≤10^7^ CFU/g feces, were omitted from further analysis because of low and unreliable read counts, although significant LDA scores were observed (some bacteria are not shown due to insufficient amounts of reads).

To determine the variations in gut microbiota composition between the acute and non-acute phases, linear discriminant analysis (LDA) coupled with effect size measurements (LEfSe) was applied to determine which taxa were enriched in the different groups according to metagenomic analysis (see detailed parameters in Materials and Methods). LEfSe determines which features (organisms, clades, operational taxonomic units, genes, or functions) are most likely to explain differences between classes by coupling standard tests for statistical significance (between the acute and non-acute phases in this study) with additional tests of biological consistency and effect relevance (Segata et al., 2011). The obtained metagenomic reads were classified at the genus level (Figure 3). Although LEfSe revealed that the genera *Rothia* and *Staphylococcus* were the most abundant during the acute phase, this dominance was not observed in all of the patients (Figure 3). Regardless, relatively increased numbers of *Ruminococcus, Blautia, Faecalibacterium*, and *Roseburia* bacteria were observed during the non-acute phase of KD (Figure 3), indicating that these genera was possibly related to remission in KD patients.

**Figure 4:**
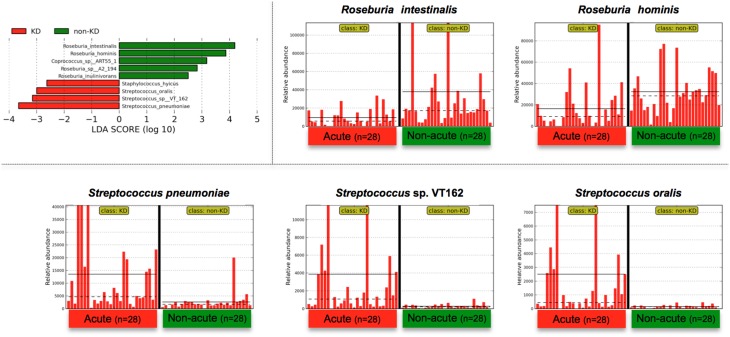
**LEfSe at the species level**. LEfSe was performed as described in Figure 3. *Roseburia* spp. were relatively abundant during the non-acute phase and could be detected at the genus level (Figure 3), whereas *Streptococcus* spp. were found to be predominant during the acute phase. Less than 0.01% of the total bacterial reads, corresponding to ≤10^7^ CFU/g feces, were omitted from further analysis because of low and unreliable read counts, although significant LDA scores were observed (*Staphylococcus hyicus* is not shown due to insufficient amounts of reads).

The above genus classifications did not reveal which common features were most likely to explain the differences between the acute and non-acute phases in the tested KD patients (*n* = 28). Because both pathogenic and non-pathogenic species may be included within one bacterial genus, we speculated that genus classifications would not reveal certain potential pathogens involved in KD pathogenesis; thus, further taxonomic classifications at the species level may allow for effective detection of KD-related pathogens (Figure 4). *Roseburia* spp. were relatively abundant during the non-acute phase and could be identified at the genus level (Figure 3), whereas *Streptococcus* spp. were predominantly identified during the acute phase, suggesting that some *Streptococcus* spp., including *S. pneumoniae, S. oralis* and other strains, are candidate KD-related pathogens (Figure 4). *Staphylococcus hyicus* might have been misidentified due to an insufficient amount of reads (less than 0.01% of the population; <1000 of the assigned reads), although the LDA score was significant.

**Figure 5:**
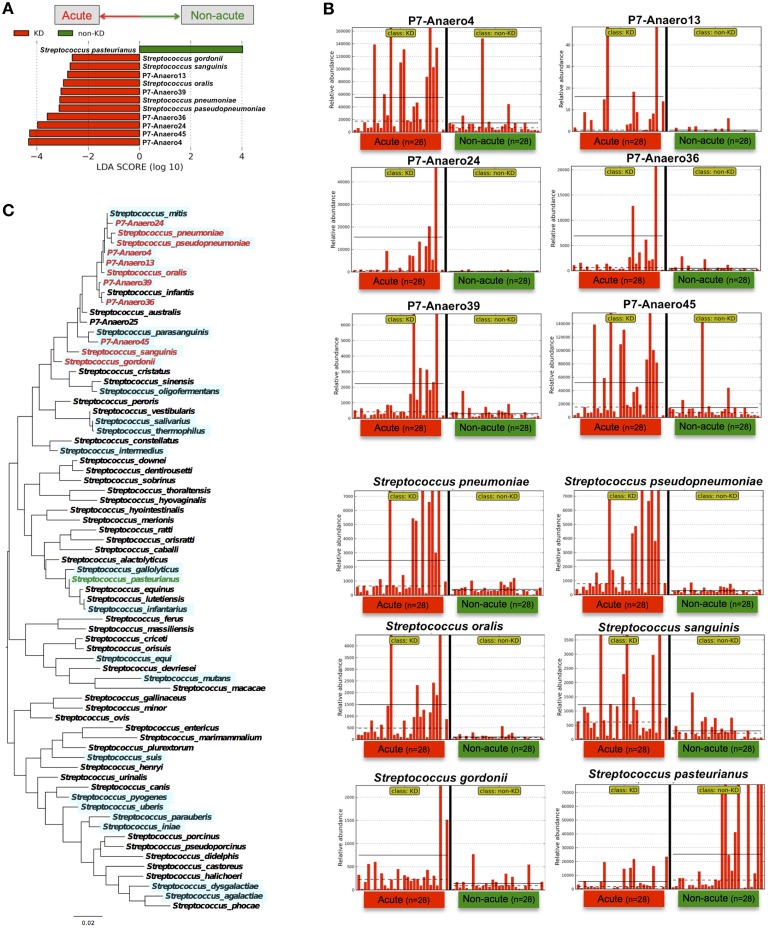
**LEfSe of P7-related ***Streptococcus*** isolates performed using ***Streptococcus*** genome database. (A)** The type of *Streptococcus* spp. markedly differed between the acute and non-acute phases of KD. **(B)** The relative abundance of detected reads for the patients (*n* = 28) was plotted for each *Streptococcus* spp. The horizontal straight line indicates the group means, and the dotted line indicates the group medians in the panel. **(C)** Maximum likelihood phylogenetic analysis of *Streptococcus* 16S-rDNA. The *Streptococcus* species in red and green are those with increased abundance during the acute and non-acute phases of KD, respectively. The complete and draft genome sequences used for the megaBLAST search are highlighted with a light blue background.

Indeed, *Streptococcus* spp. were highly abundant in the gut microbiotas of some of the KD patients; for example, 77% of the bacterial reads of one KD patient (the acute phase in P7) were from *Streptococcus*. However, the above-mentioned *S. pneumoniae* and *oralis* species were not cultured from feces during the acute phase of KD under conventional aerobic cultivation on a phenylethyl alcohol agar plate with 5% sheep blood in screening for *Streptococcus* spp., while anaerobic cultivation on chocolate agar resulted in positive *Streptococcus* colonies. In fact, fifty colonies of *Streptococcus* spp. were isolated from P7-feces on chocolate agar under anaerobic conditions with incubation at 37°C for 16 h, and then 16S-rDNA sequencing was performed to determine the bacterial species present. The results suggested that seven *Streptococcus* spp. were unique isolates (P7-Anaero4, P7-Anaero13, P7-Anaero24, P7-Anaero25, P7-Anaero36, P7-Anaero39, and P7-Anaero45) (Figure 5C). Using the draft genome sequences of seven P7-*Streptococcus* isolates, including publicly available *Streptococcus* spp. complete genomes (23 species), the metagenomic short reads of all 56 fecal samples were classified at the species level by a megaBLAST search and LEfSe. The results suggested that the amounts of the six P7-feces-related *Streptococcus* isolates (P7-Anaero4, 13, 24, 36, 39, and 45, but not P7-Anaero25) and the five detected *Streptococcus* spp. (*S. pneumonia, pseudopneumoniae, oralis, gordonii*, and *sanguinis*) were apparently increased during the acute phase in most of the KD patients, including P7, whereas *S. pasteurianus* was increased during the non-acute phase (Figures 5A,B). Intriguingly, the top 4 most abundant positive isolates were P7-feces-related *Streptococcus* spp. (P7-Anaero4, 45, 24, and 36) rather than defined pathogenic *Streptococcus* species, and all positively detected *Streptococcus* spp. were classified within a taxonomic lineage closely related to *S. oralis* or *pneumonia* (Figure 5C). All P7-feces-related *Streptococcus* isolates showed susceptibility to most antimicrobial agents, including cephem, indicating that the detection of abundant P7-feces-related isolates was most likely not correlated with antimicrobial selection.

Based on species-level identification, the first and second highest hits with identical BLAST scores constituted 17.9% of all of the short reads, which were mostly homologous to rRNA genes; thus, 82.1% of the short reads could be assigned to a unique top hit. Although the obtained 126-mer short reads might not have been sufficiently long for correct species assignments, the BLAST search results suggested that the above-mentioned P7-related *Streptococcus* groups were highly abundant during the acute phase of KD, in contrast with other pathogenic *Streptococcus* spp., such as *S. pyogenes, dysgalactiae, mutans*, and *pasteurianus*. The significant detection of unique isolates in KD patients implies a possible association of KD with uncharacterized *Streptococcus* spp.

## Discussion

Various bacterial and viral agents have been reported to be associated with KD pathogenesis (Johnson and Azimi, 1985; Catalano-Pons et al., 2005; Wang et al., 2005; Joshi et al., 2011), but these speculations have been controversial (Wang et al., 2005). Colonization by normal microbiota variants have been suggested to induce a dysregulation in the immune systems of children with a pre-existing genetic defect in immune maturation, leading to a hyperimmune reaction and the development of KD (Lee et al., 2007). In this study, the possible pathogens detected in the KD patients varied for each individual patient; thus, every identified pathogen represented a potential candidate. Regarding virus species, human adenovirus (HAdV) species F was detected in one out of twenty-eight of the patients, despite the absence of gastrointestinal manifestations in that patient. Thus, HAdV was not commonly detected, and no sequences from either other viruses or previously reported pathogens were detected in any of the other KD samples.

Although the gut microbiota markedly differed at the genus level between the acute and non-acute phases of KD (Figure 3), we speculated that classification at the species level might be appropriate for identifying disease-associated bacteria because a genus includes species that have varying effects on human health [e.g., *S. pyogenes* infections include acute rheumatic fever, pharyngitis, impetigo and streptococcal toxic shock syndrome (STSS); *S. pneumoniae* causes many types of pneumococcal infections other than pneumonia; and *S. mutans* is a significant contributor to tooth decay in the human oral cavity].

The findings of this study suggested that notable *Streptococcus* spp. in the mitis group, including *S. pneumonia, pseudopneumoniae, mitis, oralis, gordonii*, and *sanguinis*, were highly abundant in the fecal samples during the acute phase (Figures 4, 5); therefore, members of the mitis group of *Streptococci* could be present in the bacterial flora of KD patients. The mitis group comprises agents that contribute to oral biofilms, dental plaques, and infective endocarditis, disease processes that involve bacteria-bacteria and bacteria-host interactions (Whatmore et al., 2000). To further elucidate the association between *Streptococcus* spp. and KD in this study, we isolated a unique *Streptococcus* spp. (Figure 5C) and then performed whole-genome sequencing and a megaBLAST homology search. The results revealed a significant abundance of KD-derived *Streptococcus* isolates during the acute phase of the disease (Figure 5). Intriguingly, a recent paper has reported metagenomic analysis of the human gut microbiome in liver cirrhosis patients, suggesting that oral commensals, including *Streptococcus* spp., invade the gut in patients with liver cirrhosis (Qin et al., 2014) and implying that uncharacterized *Streptococcus* spp. could be potential biomarkers/pathogens for diseases with unknown etiologies.

A SAg hypothesis for KD on the etiology remains inconclusive, the involvement of single or multiple SAgs on T-cell Vβ repertoires has been speculated for the KD pathogenesis (Matsubara and Fukaya, 2007). A number of studies have found primarily Vβ2 expansion (Abe et al., 1992; Leung et al., 1995; Yoshioka et al., 1999) linking to the Vβ2 specific SAg such as toxic shock syndrome toxin-1 (TSST-1) and SpeC (Nur-Ur Rahman et al., 2011), although there is no direct evidence to suggest SAg involvement. STSS is significantly more frequent in group A ß-hemolytic streptococcal (GAS) patients than in groups B, C, and G streptococcal patients. GAS produces a multitude of surface-bound and secreted virulence factors causing resistance to phagocytosis, complement deposition, antibody opsonization, and neutrophil killing mechanisms, leading to overactive immune response and subverting host innate immune defenses (Walker et al., 2014). Although the isolation of SAg-positive *Streptococcus* from KD patients has been reported (Nagata et al., 2009; Leahy et al., 2012), group A ß-hemolytic streptococcal (GAS) might not contribute to the pathogenesis of this disease because a rapid antigen detection test (RADT) and proper antibiotic treatment prevents GAS pharyngitis during the initial episodes of acute rheumatic fever (Gerber et al., 2009). Because the KD patients in this study (Table 1) were empirically treated with antibiotics during the early stages of the disease, the results may reflect the effects of the antibiotic therapy. To address this issue, a full comparison of KD patients who have and have not received antibiotic therapy should be performed in a large, controlled study. It is also possible that antibiotic therapy has an adverse effect on the pathogenesis of KD. Further investigation of the role of *Streptococcus* spp. in the pathogenesis of KD is therefore merited.

Our previous metagenomic approach indicated that *Streptococcus* spp. were present in the lymph node specimen of one KD patient, highlighting the possible role of these bacteria in KD (Katano et al., 2012). To identify the SAg homologs, all short reads and coding sequences of the 7 isolates (P7-Anaero4, 13, 24, 25, 36, 39, and 45) were subjected to a PSI-BLAST homology search against “superantigen, staphylococcal/streptococcal toxin, bacterial (IPR013307)” orthologous proteins; however, no significant match has been found thus far (data not shown). In addition, some sequences from each sample were classified as “Not assigned” in metagenomic analysis, and new pathogenic agents remain to be characterized for some of these unidentified sequences.

The gut microbiota in the non-acute phase of KD (the distant phase) was similar in each patient, and the genera *Ruminococcus, Roseburia* and *Faecalibacterium* were predominant (Figure 3). In previous reports, an observed elevation in LPS-binding neutrophils or plasma proteins has been observed, suggesting that LPS infusion followed by disruption in intestinal mucosal barrier function might be involved in the pathogenesis of this disease (Takeshita et al., 1999, 2002b); therefore, a well-balanced commensal gut microbiota contributes to the mucosal barrier function of the intestine. Prebiotics, probiotics, and combination synbiotics modulate the balance of the intestinal microbiota and may help to prevent the onset of KD to improve patient prognosis (Bosscher et al., 2009).

The microbiota of KD patients was comprehensively analyzed in this study. Our findings suggest that markedly increased amounts of *Streptococcus* spp. are present in the gut microbiotas of acute-phase KD patients and that this difference in microbiota composition might be related to KD pathogenesis.

## Author contributions

AK and HH collected clinical specimens from the KD patients. TS, KK, and AY performed the metagenomic sequencing and statistical and bioinformatics analyses. AK and MK participated in the design of the study, performed statistical analysis, and drafted the manuscript. All authors read and approved the final manuscript.

### Conflict of interest statement

The authors declare that the research was conducted in the absence of any commercial or financial relationships that could be construed as a potential conflict of interest.

## Abbreviations

BLAST: Basic Local Alignment Search Tool
BWA-SW: Burrows-Wheeler Aligner's Smith-Waterman Alignment
CFU: colony forming unit
EDTA: ethylenediaminetetraacetic acid
FASTQ: a text-based format for storing both a biological sequence (usually a nucleotide sequence) and its corresponding quality scores
KD: Kawasaki disease
LPS: lipopolysaccharide
LEfSe: linear discriminant analysis (LDA) coupled with effect size measurements
LDA: linear discriminant analysis
MEGAN: MEtaGenome Analyzer
PCA: Principal component analysis
PERMANOVA: Permutational Multivariate Analysis Of Variance
TCR: T-cell receptor.
